# First report of *Rotylenchulus reniformis* infecting turmeric in Vietnam and consequent damage

**DOI:** 10.21307/jofnem-2020-053

**Published:** 2020-05-18

**Authors:** Huu Tien Nguyen, Quang Phap Trinh, Thi Duyen Nguyen, Wim Bert

**Affiliations:** 1 Institute of Ecology and Biological Resources, Vietnam Academy of Sciences and Technology, 18 Hoang Quoc Viet, Cau Giay, 100000 Hanoi, Vietnam; 2 Graduate University of Science and Technology, Vietnam Academy of Sciences and Technology, 18 Hoang Quoc Viet, Cau Giay, 100000 Hanoi, Vietnam; 3 3 Nematology Research Unit, Department of Biology, Ghent University, K. L. Ledeganckstraat 35, 9000 Ghent, Belgium

**Keywords:** Central Highlands, Medicinal plants, Plant-parasitic nematodes, Yield loss

## Abstract

Turmeric (*Curcuma longa* L.) is one of the common medicinal crops of high economical value in Vietnam. A survey in the Central Highlands of Vietnam revealed a turmeric growing area showing serious disease symptoms, including stunting of the plant, yellowing or darkening of the leaf margins and tips, and underdeveloped dry and rotten rhizomes. An inspection for plant pathogens in soil samples from this area revealed a high density and frequency of *Rotylenchus reniformis*, with a significant relationship between the density of *R. reniformis*, rhizome weight, and level of plant damage. This study provides the first report of *R. reniformis* found in parasitic association with turmeric in Vietnam with the support of molecular data and examines its resulting damage.

Turmeric (*Curcuma longa* L.) is a highly valuable medicinal plant, widely used as a cooking spice or for various medicinal purposes. Studies on the nutritional value of turmeric have shown the presence of many useful compounds with numerous useful medicinal properties including anti-inflammatory, antidiabetic, hepatoprotective, neuroprotective, chemoprotective, anticancer, anti-allergic, and anti-dermatophytic effects (Salehi et al., 2019). In addition, many studies have reported the usefulness of turmeric in treating different ailments such as gastrointestinal diseases, biliary and hepatic disorders, diabetic wounds, rheumatism, inflammation, sinusitis, anorexia, coryza, and cough (Ammon et al., 1992; Nasri et al., 2014; Salehi et al., 2019). Thanks to these valuable properties, turmeric is widely cultivated in many regions in Vietnam, such as Lao Cai, Lang Son, Vinh Phuc, Hung Yen, Nghe An, and the Central Highlands, where it represents a crop of high economic significance to farmers. In order to achieve a sustainable development of turmeric in these regions, it is vital that comprehensive plant-pathogen management strategies can be formulated and put into practice, including the management of plant-parasitic nematodes (Sikora et al., 2018). A number of nematode species have been reported associated with turmeric world-wide (Sikora et al., 2018; CABI, 2019), and *Meloidogyne incognita* and *Rotylenchus reniformis* in particular are known to be widely distributed and to cause significant damage to turmeric crops (Mani et al., 1987; Routaray et al., 1987; Haider et al., 1998). To the best of our knowledge, studies of pathogens associated with turmeric in Vietnam is very limited, especially for plant-parasitic nematodes. The reports of *M. incognita* and *M. javanica* in Vietnamese turmeric are the only studies that can be found on the subject (Vu et al., 2018; Le, 2019). It is therefore vital to implement further study on plant-parasitic nematodes in Vietnam using an integrated approach, and to evaluate their damage potential to turmeric crops in order to provide a basis for pest management strategies.

## Material and methods

Soil samples were collected following a grid pattern method from a turmeric growing area in the Central Highlands of Vietnam. At 10 sampling sites a visual inspection of plant symptoms was combined with the collection of four samples around a clump of turmeric, after removing the detritus layer to create a bulk sample (collection of 40 soil samples using a core (5 × 25 cm) resulted in 10 bulk samples). Percentage of yellowing leaves, dry rot rhizomes, and rhizome weight for each clump of turmeric were recorded (Coyne et al., 2018). Permanent slides of nematodes were made following Nguyen et al. (2019a). Morphological identification was done based on Robinson et al. (1997) and Dasgupta et al. (2011). For molecular identification, D2-D3 regions of 28 S rDNA and *COI* mtDNA sequences were amplified and analyzed following Nguyen et al. (2019b). The correlations between the density of *R. reniformis* and any plant damage (yellowing leaves and dry rot rhizome), as well as rhizome weight over all sampling sites was analyzed using SPSS version 25 after checking data assumptions (IBM Corp, 2017).

## Results and discussion

The inspection of soil samples from the turmeric growing area revealed a high frequency of *R. reniformis* (90%) at a relatively high density (up to 480 nematodes/100 ml of soil). Morphological characterizations of *R. reniformis* in this study are in agreement with the description of *R. reniformis* by Robinson et al. (1997) and Dasgupta et al. (2011), including body length of immature females (409 ± 21 (380−430) μm), stylet length (16.5 ± 0.8 (15−17) μm), vulva position (71 ± 2 (69−74)%), and presence of males. The D2-D3 of 28 S rDNA (accession number: MT225542) and *COI* mtDNA (accession number: MT232760, MT232761) sequences of our population were respectively 99.5 and 100% similar to the most closely-related D2-D3 of 28 S rDNA sequences (accession number: KT003743-KT003745, HM131858) and *COI* mtDNA sequences (accession number: MK908054, MK908055, LC348942−LC348948, KT003730) of *R. reniformis* from GenBank.

**Figure 1:**
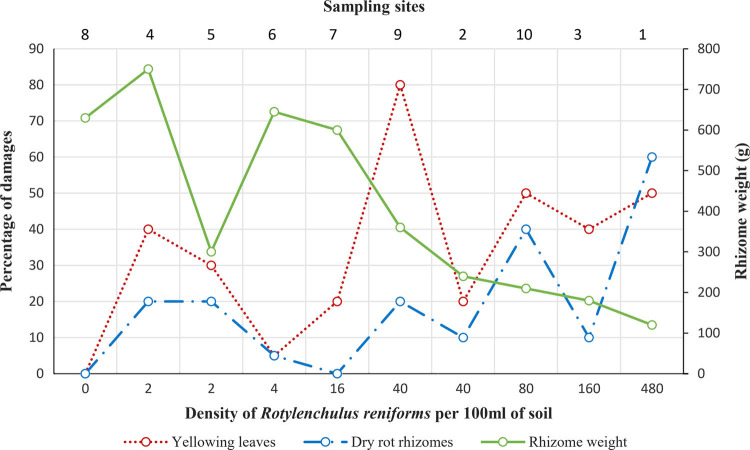
Graph showing the relationship between the density of *R. reniformis* and rhizome weight, and percentage of yellowing leaves and dry rot rhizomes.

A strong correlation between the density of *R. reniformis* and rhizome weight was found (R_S_ = −0.841, Spearman correlation test, significance level = 0.002). The relation of nematode density and damage (yellowing leaves and dry rot rhizome) appeared to be more fluctuating (Figure 1). However, a highly significant correlation between the density of *R. reniformis* and the amount of plant damage (yellowing leaves and dry rot rhizomes) was found (R_S_  = 0.64, Spearman correlation test, significance level = 0.046 and R = 0.781, Pearson correlation test, significance level = 0.008, respectively) (Table 1).

**Table 1.** Correlation coefficient between the density of *R. reniformis*, the percentage of yellowing leaves, dry rot rhizomes, and rhizome weight.

Correlations	*R. reniformis*	Yellowing leaves	Dry rot rhizome
*Yellowing leaves*			
Pearson (R_P_)	0.355		
Spearman (R_S_)	0.640*		
*Dry rot rhizome*			
Pearson (R_P_)	0.781**	0.601	
Spearman (R_S_)	0.506	0.838**	
*Rhizome weight*			
Pearson (R_P_)	−0.623	−0.480	−0.600
Spearman (R_S_)	−0.841**	−0.502	−0.519

**Notes:** *,**Significant at 0.05 and 0.01 levels (2-tailed), respectively.

**Figure 2:**
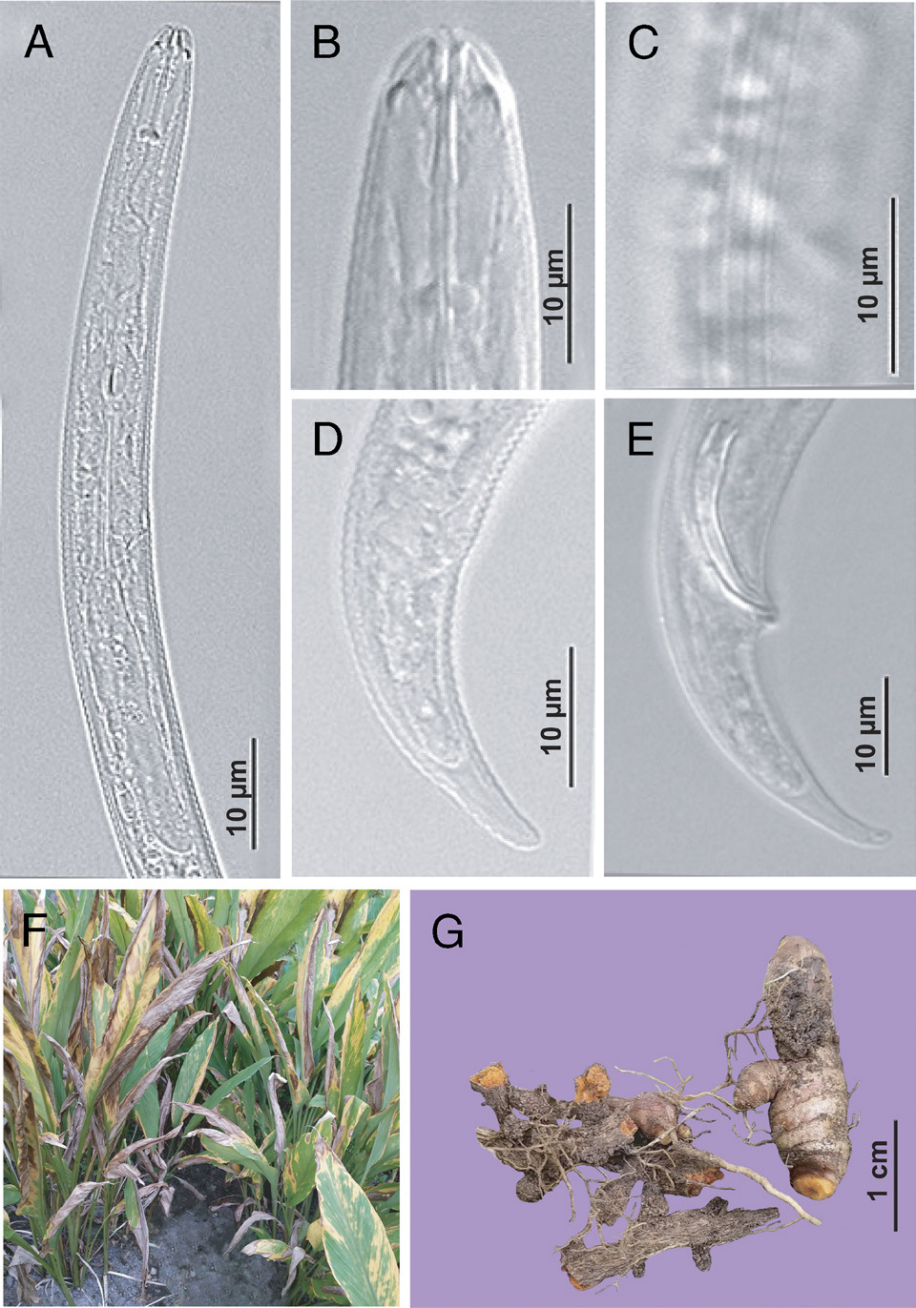
LM pictures of *R. reniformis* and disease symptoms of turmeric in studied growing area. A−D: Immature female. A: Pharyngeal region; B: Labial region; C: Lateral field; D: Tail region of immature female; E: Tail region of male; F: Aerial part; G: Underground part.

Nair (2019) reported that plant-parasitic nematodes such as *M. incognita*, *Pratylenchus coffeae*, and *Radopholus similis* can cause stunting, discolouration, and rotting of mature rhizomes in turmeric − symptoms that are indeed similar to those recorded in this study (Figure 2). It is well known that *R. reniformis* is one of the most devastating plant-parasitic nematodes in the world (Robinson et al., 1997; Jones et al., 2013), even at densities between 0.1 to 5 nematodes/1 cm^3^ of soil (Gaur and Perry, 1991). Furthermore, *R. reniformis* is reportedly able to deplete the roots of certain plants and facilitate the infection of different harmful fungi (Lele et al., 1978; Taha and Kassab, 1980; Tchatchoua and Sikora, 1983). Remarkably, for turmeric, Haider et al. (1998) found that *R. reniformis* caused a significantly higher growth reduction compared to the infamous damaging root-knot nematode *M. incognita*. In our study, the relatively high density of *R. reniformis* (up to 480 nematodes/100 ml of soil) and the significant correlations between *R. reniformis* and the level of plant damage (yellowing leaves and dry rotten rhizomes) indicate the clear involvement of *R. reniformis* in causing plant damage and yield loss of turmeric in the Central Highlands of Vietnam. Although *R. reniformis* has already been widely reported in several other crops in Vietnam such as banana and coffee (Nguyen and Nguyen, 2000; Trinh et al., 2009), these reports were unsubstantiated by molecular data or damage assessment (Nguyen and Nguyen, 2000; Trinh et al., 2009). The present study provides the first report of *R. reniformis* associated with turmeric in Vietnam with the support of molecular data, which is vitally necessary given the presence of cryptic species in *Rotylenchulus* (Singh *et al.*, 2020, in review). Furthermore, the observed relation of nematode density to rhizome biomass and disease symptoms in this study confirms the importance of this nematode as a pest menace to turmeric. Despite the many experiments that have been done in lab conditions to estimate the impact of *R. reniformis* on crops over the world (Robinson et al., 1997), damage assessments using different approaches under field conditions are also of crucial importance to provide real-life, up-to-date information and datasets that will contribute in a meaningful way to forming a basis for nematode management strategies both in Vietnam and worldwide.
